# Puncturing the Bladder above the Pubis

**Published:** 1873-10

**Authors:** W. C. Raymond

**Affiliations:** Cambria, Niagara Co., N. Y.


ART. III.—*Puncturing the Bladder above the Pubis.* By W. C. RAYMOND, M. D., Cambria, Niagara Co., N. Y.

It is generally conceded I believe, that "Puncturing the Bladder above the Pubis" is seldom necessary and that only in cases of enlarged prostate complicated with impermeable stricture, and but seldom performed. Hence my apology for reporting the subjoined case as its favorable termination might induce for it a further trial.

Was called July 17th, 1873, to see Leverett Sperry, aged 70 years and three months, an American, occupation a farmer, of spare habit, subject for many years to attacks of Asthma, suffering from retention of urine of thirty-six hours' standing from strictures and enlarged prostate.

I succeeded without much difficulty in introducing a large sized Gum Elastic Catheter, although the strictures seemed quite strong, and relieved the bladder of nearly a quart of urine, repeated the catheterization the next morning, after which time the patient passed the instrument himself until the evening of the 20th of July, when I was again summoned, found him suffering from retention and, although I could pass the cathether the whole length without difficulty, it did not relieve the bladder but was attended with considerable hemorrhage.

Judged that he had in his haste and suffering passed the instrument as far as one of the pockets, or folds of the urethra when by force he pushed it on and formed an artificial passage.

I concluded to tap the bladder, above and at point close down to the pubis, below where the peritoneum is reflected over the bladder, which I did with a lancet, making a free external opening I passed the lancet directly into the bladder, introducing a Gum Elastic Catheter of considerable length, which enabled me by depressing the end in the form of siphon to relieve the bladder of about pint and a half of decomposed blood, mucus and urine, when the patient expressed himself as feeling pretty well, not having suffered very much from the shock of operation, notwithstanding he had taken no anæsthetic.

Rested well through the night. Visited him next day, washed out the bladder with warm water, gave mucilaginous drinks with mild diet, washed out the bladder daily. At the end of a week introduced the catheter through the urethra and evacuated the bladder, and he now passes water both through the natural and artificial openings. He strenously objects, having the artificial opening closed, as it might call for a repetition of the operation some time.

He expresses himself as being quite as well now as he had been for months before the operation.

